# TESTING THE IMPLEMENTATION FRAMEWORK FOR BEHAVIORAL AND LIFESTYLE INTERVENTIONS IN ALZHEIMER’S DISEASE (MOBILIZE)

**DOI:** 10.1093/geroni/igae098.3034

**Published:** 2024-12-31

**Authors:** Dereck Salisbury, Vankee Lin, Fang Yu

**Affiliations:** University of Minnesota, University of Minnesota, Minnesota, United States; Stanford University, Stanford, California, United States; Arizona State University, Phoenix, Arizona, United States

## Abstract

**Introduction:**

Implementing multi-site behavioral intervention trials in Alzheimer’s disease (AD) has many unique challenges, leading to substantial variations in delivered intervention doses and cognitive findings. These issues can be addressed by the IMplementation Framework fOr Behavioral and LIfestyLe Interventions In AlZheimer’s DiseasE (MOBILIZE) that was developed to guide the design and implementation of behavioral interventions in AD.

**Methods:**

This study systematically evaluated the implementation outcomes of the multi-site aerobic exercise and cognitive training (ACT) trial that employed MOBILIZE. Building on the person-centered care principle, MOBILIZE includes three implementation outcomes (screening, intervention adherence, and safety) with corresponding team processes to guide trial implementation. Screening was operationalized as the duration (days) between each screening visit, last screening visit to enrollment, and enrollment to first intervention session. Intervention adherence outcomes included: adherence (attendance), session dose adherence (calculated by dividing the number of sessions that achieved >=70% of the prescribed session dose / total number of sessions completed), and days required to complete the prescribed 72 sessions. Safety was operationalized as the type, number, and severity of study-related adverse events (AEs). RESULTS. The sample (n=146) was 73.8±5.7 years in age and 23.4±2.1 on Montreal Cognitive Assessment score, with 48.0% female, and 91.8% white. Screening-to-enrollment averaged 117.4±71.7 days. Intervention adherence was 77.4%±28.3%. There was 9-study related adverse events. Screening-to-enrollment and intervention adherence differed across sites, mainly due to COVID-19 influence. CONCLUSIONS. MOBILIZE helped the ACT Trial achieve high intervention adherence and safety and may be particularly important for early-stage and multi-site trials in AD.

